# Horizontal gene transfer of a chloroplast DnaJ-Fer protein to Thaumarchaeota and the evolutionary history of the DnaK chaperone system in Archaea

**DOI:** 10.1186/1471-2148-12-226

**Published:** 2012-11-26

**Authors:** Céline Petitjean, David Moreira, Purificación López-García, Céline Brochier-Armanet

**Affiliations:** 1 UPR CNRS 9043, Laboratoire de Chimie Bactérienne, Université d’Aix-Marseille (AMU), 13402 Marseille, Cedex 20, France; 2 UMR CNRS 8079, Unité d'Ecologie, Systématique et Evolution Université Paris-Sud, 91405 Orsay, Cedex, France; 3 CNRS, UMR5558, Laboratoire de Biométrie et Biologie Evolutive, Université de Lyon, Université Lyon 1, 43 boulevard du 11 novembre 1918, 69622, Villeurbanne, France

**Keywords:** DnaJ/Hsp40, DnaK/Hsp70, Hyperthermophily, Archaeplastida, Phylogeny, Archaea, Thaumarchaeota, Horizontal gene transfer, Mesophily

## Abstract

**Background:**

In 2004, we discovered an atypical protein in metagenomic data from marine thaumarchaeotal species. This protein, referred as DnaJ-Fer, is composed of a J domain fused to a Ferredoxin (Fer) domain. Surprisingly, the same protein was also found in Viridiplantae (green algae and land plants). Because J domain-containing proteins are known to interact with the major chaperone DnaK/Hsp70, this suggested that a DnaK protein was present in Thaumarchaeota. DnaK/Hsp70, its co-chaperone DnaJ and the nucleotide exchange factor GrpE are involved, among others, in heat shocks and heavy metal cellular stress responses.

**Results:**

Using phylogenomic approaches we have investigated the evolutionary history of the DnaJ-Fer protein and of interacting proteins DnaK, DnaJ and GrpE in Thaumarchaeota. These proteins have very complex histories, involving several inter-domain horizontal gene transfers (HGTs) to explain the contemporary distribution of these proteins in archaea. These transfers include one from Cyanobacteria to Viridiplantae and one from Viridiplantae to Thaumarchaeota for the DnaJ-Fer protein, as well as independent HGTs from Bacteria to mesophilic archaea for the DnaK/DnaJ/GrpE system, followed by HGTs among mesophilic and thermophilic archaea.

**Conclusions:**

We highlight the chimerical origin of the set of proteins DnaK, DnaJ, GrpE and DnaJ-Fer in Thaumarchaeota and suggest that the HGT of these proteins has played an important role in the adaptation of several archaeal groups to mesophilic and thermophilic environments from hyperthermophilic ancestors. Finally, the evolutionary history of DnaJ-Fer provides information useful for the relative dating of the diversification of Archaeplastida and Thaumarchaeota.

## Background

The 70 kD heat shock proteins (called DnaK in bacteria and Hsp70 in eukaryotes) form a large family of molecular chaperones upregulated in cells suffering various stresses, including heat shocks and heavy metal exposure [1,2]. In addition, these proteins play a major role during protein synthesis by binding to the nascent peptides exiting the ribosome in order to prevent their aggregation and facilitating their folding in the optimal functional conformation [3]. During the interaction with the partially synthesized peptides, DnaK/Hsp70 increases its ATPase activity [3]. This chaperone has two main partners: the J-proteins [4,5] and the nucleotide exchange factor, called GrpE in bacteria (or Mge1 [6] in mitochondria and Cge1 [7] in chloroplasts) and Bag-1, a eukaryotic functional analogue of GrpE [8]. The nucleotide exchange factor promotes the exchange of ADP to fresh ATP in the nucleotide-binding region of DnaK/Hsp70, whereas the J-proteins stimulate the ATPase activity in order to stabilize the interaction of DnaK with unfolded proteins [5,9,10]. The J-proteins form a large family of proteins, which are structurally and functionally diverse but all have the capacity to interact with DnaK/Hsp70 through their J-domain [4,11]. Among them, DnaJ/Hsp40 proteins form the largest subfamily [12]. They control the flux of unfolded polypeptides into and out of the substrate-binding domain of DnaK/Hsp70 [9,11].

DnaK proteins are widespread, being encoded by a single gene in most bacterial genomes, whereas most eukaryotic genomes harbor several Hsp70 genes that may have diverse evolutionary origins [1,13,14]. For example, in the green alga *Chlamydomonas reinhardtii*, five Hsp70 copies are present, all them encoded in the nuclear genome despite being targeted in diverse cellular compartments: three of them most likely originated by duplications from an ancestral eukaryotic gene (one expressed in the cytoplasm and two in the endoplasmic reticulum); one has a mitochondrial origin and is exported into the mitochondria, whereas the latter originated from the chloroplast endosymbiosis and is targeted into the chloroplast [15]. In contrast with DnaK, the J-proteins are encoded in multiple copies in bacterial genomes [9]. This is also the case in eukaryotes, where they work in the different cell compartments in association with the Hsp70 proteins cited above [9,11]. Finally, the nucleotide exchange factor GrpE is present in one copy in most of bacterial genomes, whereas the eukaryotic Mge1, Cge1 and Bag-1 are encoded in the nucleus but addressed to the mitochondria, chloroplasts, and to the nucleus and the cytoplasm, respectively [7,8].

The presence of DnaK, DnaJ and GrpE has been reported in several archaeal genomes [16], more precisely in several euryarchaeota but never in crenarchaeotal species. The best studied case concerns DnaK. A phylogenetic analysis by Gribaldo and coworkers suggested that this protein was acquired by several archaea by horizontal gene transfer (HGT) from different bacterial donors [17]. These authors observed three different groups of archaeal DnaK sequences branching specifically with certain bacterial homologues. More precisely, *Methanosarcina mazei* (Methanosarcinales) was related to the *Clostridium* group of Firmicutes (low G+C Gram positive bacteria), *Halobacterium cutirubrum* and *Halobacterium marismortui* (Halobacteriales) to the Actinobacteria (high G+C Gram positive bacteria), whereas *Methanobacterium thermautotrophicum* (Methanobacteriales) and *Thermoplasma acidophilum* (Thermoplasmatales) branched with *Thermotoga maritima* (Thermotogales) [17]*.* More recently, Macario *et al.* (2006) studied in various bacteria and archaea the taxonomic distribution and the phylogeny not only of DnaK but also of GrpE and DnaJ. They showed that the genes coding for these three proteins were clustered in most of the genomes examined [16]. They also confirmed the results of Gribaldo *et al.* (1999), i.e. the likely existence of three HGT events from bacteria to archaea. However, they proposed a more complex scenario where the DnaK/DnaJ/GrpE cluster was first acquired from a bacterial donor by the ancestor of the Euryarchaeota, then lost in Methanococcales and in the common ancestor of Archaeoglobales, Halobacteriales and Methanosarcinales, and finally reacquired independently by Halobacteriales and Methanosarcinales from Actinobacteria and from Firmicutes, respectively [16]. Worth noting, in these two studies, none of the three proteins was detected in hyperthermophilic archaea.

In addition to these relatively well-characterized chaperones and co-chaperones, the study of a genomic fragment of an uncultured deep marine archaeon from an environmental DNA fosmid library revealed a very unusual J-protein, referred as DnaJ-Fer, composed of a J-domain fused with a Ferredoxin (Fer) domain [18]. The phylogenetic analysis of a 16S rRNA gene also found in this genomic fragment showed that it belonged to a member of the Thaumarchaeota, more precisely in the I.1a subgroup. These archaea, formerly classified as Group I, a sublineage of Crenarchaeota [19,20], have been recently proposed to represent a third phylum of Archaea together with the Euryarchaeota and Crenarchaeota [21]. Thaumarchaeota are widespread in many environments, including marine and freshwater, soil and sediment [22,23]. Surprisingly, the presence of DnaJ-Fer proteins has also been reported in Viridiplantae (including green algae and plants), with three homologues (CDJ3, 4 and 5) in *C. reinhardtii*[24]. These proteins are localized in the chloroplast of this green alga where they interact with the chloroplast Hsp70B and Cge1 proteins. However, the precise function of these DnaJ-Fer proteins in *C. reinhardtii* remains to be elucidated. According to the location and the nature of its partners, it would be tempting to hypothesize a cyanobacterial origin of the DnaJ-Fer protein. However, no homologue has been detected in Cyanobacteria [24].

Two hypotheses can explain the unexpected taxonomic distribution of the DnaJ-Fer protein in Thaumarchaeota and Viridiplantae: either two independent and convergent fusions of the two protein domains occurred in these two distantly related lineages, or a single fusion occurred in one of them followed by a HGT to the other lineage [24]. In this work, we have taken advantage of the recent burst of available archaeal complete genome sequences [25], including representatives of new major lineages such as the Thaumarchaeota, ARMAN or Nanohaloarchaeales, to decipher the evolutionary history of DnaK and its co-chaperones in Archaea, with especial attention on the intriguing DnaJ-Fer protein. Our results support a complex scenario in which HGT appears to have played an important role. In addition to other cases of HGT, Thaumarchaeota appear to have most likely acquired their DnaK, co-chaperones and DnaJ-Fer proteins by independent HGTs from multiple donors, including other archaea and plants.

## Results

### DnaJ-Fer proteins are widespread in viridiplantae and thaumarchaeota

We carried out an intensive survey of public sequence databases to find that DnaJ-Fer homologues are present in all Viridiplantae (green algae and land plants) for which complete genome sequences were available. In contrast, we did not detect them in Rhodophyta and Glaucophyta, the two other lineages composing the Plantae or Archaeplastida eukaryotic supergroup [26]. However, due to the scarcity of sequence data from these two lineages, we can not exclude the future discovery of DnaJ-Fer in some species belonging to them. In addition to green algae and land plants, DnaJ-Fer homologues were detected in the four available complete genomes of Thaumarchaeota (Additional file 1): *Cenarchaeum symbiosum* (a sponge symbiont) [27], the planktonic *Nitrosopumilus maritimus* (the first isolated thaumarchaeote) [28] and its two close relatives ‘*Candidatus* (*Ca*.) Nitrosoarchaeum limnia SFB1’ [29] and in ‘*Ca*. Nitrosoarchaeum koreensis MY1’ [30] which live in low salinity sediments and in the soil rhizosphere, respectively, as well as in several environmental fosmid sequences, all likely members of the mesophilic group I.1a. The protein was also present in *Nitrososphaera viennensis* (Schleper and Spang, personal communication) and ‘*Ca*. Nitrososphaera gargensis’ [31], two moderate thermophilic representatives of the group I.1b. In contrast, it was absent in the thermophilic species ‘*Ca*. Nitrosocaldus yellowstonii’, a representative of the more distant Hot Water Crenarchaeotic Group (HWCG) III (de la Torre, personal communication), and in ‘*Ca*. Caldiarchaeum subterraneum’ [32], a representative of the ‘Aigarchaeota’ (formerly group HWCG I) which seems to be either the sister group of Thaumarchaeota or a deeply branching thaumarchaeotal lineage [22].

**Figure 1:**
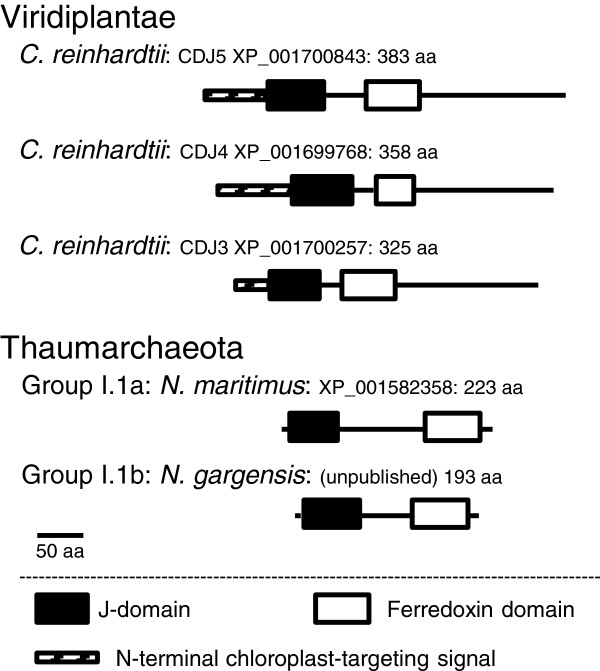
**Structural organisation of the DnaJ-Fer proteins. **The organisation of the DnaJ-Fer is shown for the three homologues found in *Chamydomonas reinhardtii* (Viridiplantae) and for the single protein present in *Nitrosopumilus maritimus *and ‘*Ca*. Nitrososphaera gargensis’ (Thaumarchaeota Groups I.1a and I.1b, respectively).

The J and Fer domains are two small domains (less than 100 amino acids) well conserved in the plant and thaumarchaeotal DnaJ-Fer sequences. The Fer domain was characterized by an amino acid motif CXXCXXC observed in all those sequences except in ‘*Ca*. N. gargensis’ and *N. viennensis*, where the motif was CXXFXXC. Contrasting with the conservation of these two domains, we observed different sequence organizations of the DnaJ-Fer proteins in the Viridiplantae and in the Thaumarchaeota (Figure 1). In Viridiplantae, an N-terminal chloroplast signal region preceded the J and the Fer domains, and the protein ended with a long C-terminal region (up to 150 amino acids) of unknown function. In Thaumarchaeota, these N- and C-terminal regions were absent, but an inter-domain region (ranging between 54 and 92 amino acids) was present between the J and the Fer domains. This region was well conserved in *N. maritimus*, *C. symbiosum*, ‘*Ca*. Nitrosoarchaeum limnia SFB1’, ‘*Ca*. Nitrosoarchaeum koreensis MY1’ and the fosmids found in the environmental database (all belonging to the group I.1a), but was divergent and shorter (54 amino acids) in the sequences of ‘*N. gargensis*’ and *N. viennensis*, the two representatives of group I.1b. The presence of this variable central region suggested that its role is probably structural and not functional in Thaumarchaeota. By contrast, much shorter or no central regions were present between the two domains in the plant sequences.

### The taxonomic distribution of the DnaJ-Fer protein results from an ancient HGT

**Figure 2:**
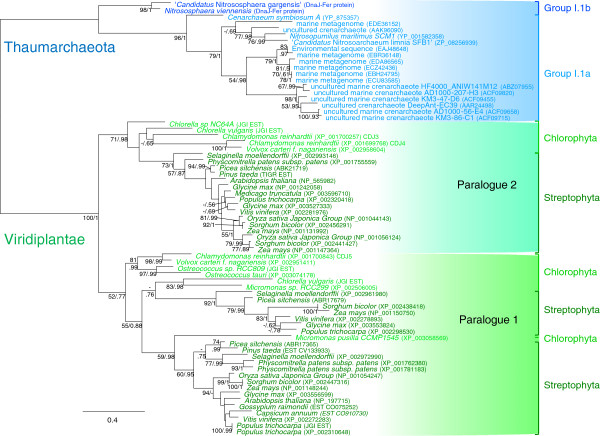
**Unrooted ML phylogenetic tree of the DnaJ-Fer protein. **The tree was reconstructed with 69 sequences and 127 positions with TreeFinder and the LG model + Γ4. Numbers at nodes represent bootstrap values and Bayesian posterior probabilities computed by TreeFinder and PhyloBayes, respectively (only values >50% and 0.5 are shown, dashes indicate that the corresponding support is inferior to the threshold, whereas when both supports are inferior to the thresholds no support values are indicated). The scale bar represents the average number of substitutions per site. The Viridiplantae sequences are shown in green and those of Thauamrchaeota in blue.

Maximum likelihood (ML) analyses and Bayesian inference (BI) of the DnaJ-Fer alignment revealed three monophyletic groups (Figure 2). Two corresponded to Viridiplantae (ML bootstrap values (BV) = 71% and 52%, and BI posterior probabilities (PP) = 0.98 and 0.78, respectively) whereas the third gathered the thaumarchaeotal sequences (BV = 100% and PP = 1.00). Interestingly, the relationships among sequences within each of these groups were in agreement with the accepted species phylogeny and relatively well supported despite the small number positions (127 amino acids) kept for the phylogenetic analysis. More precisely, the dichotomy between group I.1a and group I.1b Thaumarchaeota was well supported (BV = 96% and PP = 1.00). The relationships among the green algae and land plant sequences were more complex since there were several copies of this protein in these species, most likely resulting from duplication events. A first duplication occurred almost certainly in the ancestor of Viridiplantae, leading to the two paralogues present in green algae and land plants. This event was followed by additional duplication events at the origin of the multiple copies of paralogues 1 and 2 observed in the viridiplantae lineages (Figure 2).

**Figure 3:**
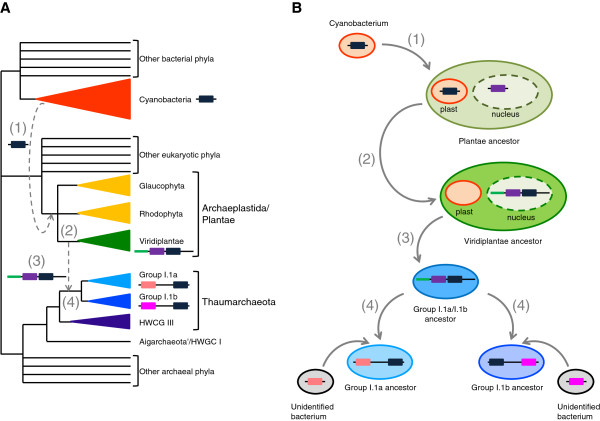
**Origin and evolution of the DnaJ-Fer protein. **(**A**) Schematic representation of the tree of life with the three Domains (Archaea, Bacteria and Eucarya) showing the time of the evolutionary events that have affected the DnaJ-Fer protein. (**B**) Evolutionary scenario for the origin and evolution of the DnaJ-Fer protein: (1) Acquisition of a cyanobacterial Fer domain-containing protein by the ancestor of Archaeplastida/Plantae; (2) translocation of the corresponding gene in the nucleus, fusion with a J domain coding gene and addition of a chloroplast signal peptide; (3) horizontal gene transfer of the DnaJ-Fer coding gene to the ancestor of thaumarchaeota groups I.1a and I.1b and (4) independent replacement of the J domain by J domains of bacterial origin in thaumarchaeotal groups I.1a and I.1b.

Phylogeny results indicated that the ancestor of thaumarchaeotal groups I.1a and I.1b already harboured the DnaJ-Fer gene and that the ancestor of Viridiplantae had two copies. If the unusual taxonomic distribution of DnaJ-Fer proteins was indicative of an HGT between Thaumarchaeota and Viridiplantae, the inferred phylogenies suggested that this HGT took place before the diversification of these two major lineages and was therefore relatively ancient (event 3 on Figure 3A). However, due to the lack of any suitable outgroup (no other lineage contained the DnaJ-Fer protein) it was not possible to determine the precise evolutionary origin of the DnaJ-Fer gene and the direction of the HGT between Thaumarchaeota and Viridiplantae. To tackle this issue we carried out phylogenetic analyses of the J and Fer domains separately. Indeed, although the association between these two domains is specific of Thaumarchaeota and Viridiplantae, each domain is widely distributed in present day organisms, opening the possibility to reconstruct rooted phylogenies for each of them.

### The J and Fer domains have two different evolutionary origins

**Figure 4:**
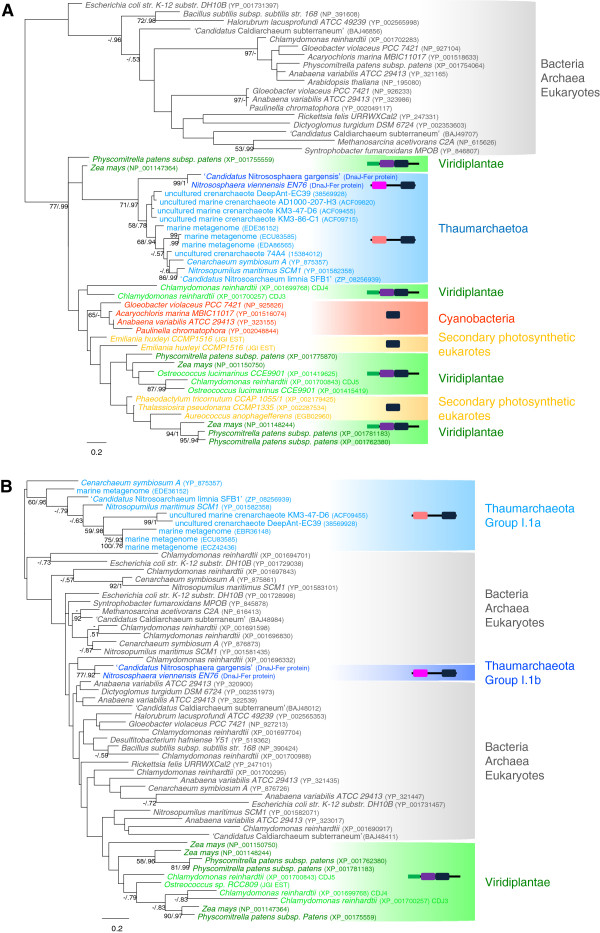
**Unrooted ML trees of the Fer and J domains. **The ML tree of the Fer domain (**A**) was inferred with 52 sequences and 41 positions, whereas 55 sequences and 40 positions were kept to reconstruct the J domain tree (**B**). The two trees were inferred with TreeFinder (LG model). Numbers at nodes represent bootstrap values and Bayesian posterior probabilities computed with TreeFinder and PhyloBayes, respectively (only values >50% and 0.5 are shown, dashes indicate that the corresponding support is inferior to the threshold, whereas when both supports are inferior to the thresholds no support values are indicated). The scale bars represent the average number of substitutions per site. For clarity, the sequences relevant for the understanding of the history of DnaJ-Fer proteins have been coloured according to their taxonomy.

As expected because of the small number of conserved sequence positions, the ML phylogeny of the Fer domain was largely unresolved (data not shown). Nevertheless, the Fer domain of the DnaJ-Fer proteins of Viridiplantae and Thaumarchaeota branched within a single cluster, which also contained various bacterial and archaeal sequences. To improve the resolution of the phylogenetic relationships between these sequences, we carried out an analysis of the sequences composing this cluster and close relatives using several more distantly related sequences as outgroup. The resulting ML tree supported the grouping of thaumarchaeotal and viridiplantae sequences (BV = 77% and PP = 0.99, Figure 4A), indicating that the Fer domain of the DnaJ-Fer proteins had a single origin and, most likely, that a HGT event occurred between these two distant lineages. Interestingly, Fer domains from cyanobacterial and stramenopile species branched in the same cluster (Figure 4A). Stramenopiles are eukaryotes that acquired a chloroplast secondarily from Rhodophyta [33]. Therefore, the grouping of viridiplantae, stramenopile and cyanobacterial sequences strongly suggested a cyanobacterial origin of the Fer domain in these two eukaryotic photosynthetic lineages, even if the sequences of the photosynthetic eukaryotes did not appear nested within the cyanobacterial sequences. In fact, this was likely due to a poor resolution of the phylogenetic tree, which is frequent in similar studies of proteins of cyanobacterial origin, where most often only a sister-grouping of cyanobacteria and plant sequences is observed in phylogenetic trees [34]. The hypothesis of an HGT from plants to cyanobacteria can be discarded because the protein is present in *Gloeobacter*, which is a deeply branching cyanobacterial lineage that has diverged before the chloroplastic endosymbiosis and, consequently, before the origin of plants [35]. The HGT of the Fer domain from cyanobacteria to plants is also strongly supported by the functional data showing that the DnaJ-Fer protein is targeted to the chloroplast in the green alga *Chlamydomonas*[24]. It is important to notice that, in contrast with the two-domain DnaJ-Fer proteins of Viridiplantae and Thaumarchaeota, the stramenopile and cyanobacterial proteins were composed uniquely of the Fer domain. Thus, the association between the J and the Fer domains probably occurred in the Viridiplantae lineage after the divergence of the present-day three main Archaeplastida phyla (i.e., Viridiplantae, Rhodophyta and Glaucophyta) but prior to the internal diversification of Viridiplantae (Figure 3A). This phylogeny also supported that the ancestor of the thaumarchaeotal groups I.1a and I.1b acquired secondarily the DnaJ-Fer protein from an ancestor of present-day Viridiplantae (Figure 3A). Another possibility would be that Viridiplantae and Cyanobacteria acquired their Fer domain from Thaumarchaeota. This would imply two HGT events, one from Thaumarchaeota to Cyanobacteria and a second one from Cyanobacteria to photosynthetic eukaryotes through the chloroplast endosymbiosis. In addition, that hypothesis would also imply the dissociation of the Fer and J domains in Cyanobacteria and their reassociation in the Viridiplantae lineage. Therefore, this scenario would require two HGTs as well as two independent associations and one split between the J and Fer domains, what is less parsimonious than the previous one that only requires one association and two HGT events.

Although poorly resolved as in the case of the phylogeny of the Fer domain, the phylogeny of the entire data set of J domain sequences yielded a very different picture. In fact, Viridiplantae and Thaumarchaeota did not cluster together, which was confirmed by a second analysis based on a more restricted sequence sampling. The J domains from the DnaJ-Fer proteins formed three distinct groups (indicated by colours in Figure 4B) scattered among J domain sequences of very different origins (bacterial, eukaryotic and archaeal) and being part of very diverse multidomain proteins. One group contained the J domains from Viridiplantae DnaJ-Fer proteins, another contained those from the group I.1b Thaumarchaeota (i.e., ‘*Ca*. N. gargensis’ and *N. viennensis*), whereas group I.1a Thaumarchaeota emerged in another part of the tree (Figure 4B). This separation in three groups suggested that the J domains of the DnaJ-Fer proteins have different origins. However, this could be due just to the overall poor resolution of the trees. Thus, to discriminate between these two hypotheses (i.e. different origins or lack of phylogenetic signal) we compared the topology of the ML tree with AU tests against four constrained topologies reflecting alternative scenarios for the origin of the DnaJ domain contained in the DnaJ-Fer proteins: 1) the grouping of the J domains of the DnaJ-Fer proteins of the two groups of Thaumarchaeota I.1a and I.1b (Topology 2); 2) the monophyly of these sequences plus the J domains of the DnaJ-Fer proteins of the Viridiplantae (Topology 3); 3) the monophyly of group I.1a Thaumarchaeota and Viridiplantae DnaJ-Fer J domains (Topology 4); and 4) the monophyly of group I.1b Thaumarchaeota and Viridiplantae DnaJ-Fer J domains (Topology 5) (Table 1), the other nodes remaining unchanged. The five topologies were used for the AU test with the alignment of J domain sequences used for the inference of the initial topology (Topology 1). All the four alternative topologies were significantly rejected (p<0.05, Table 1), which indicated that the J domains found in the DnaJ-Fer proteins probably have three independent evolutionary origins.

**Table 1:** AU tests of scenarios for the origin of the J domain contained in DnaJ-Fer proteins

**Topology**	**Scenario**	***p*-value**
1	Viridiplantae, Group I.1a and Group I.1b not monophyletic	0.991
2	Group I.1a and Group I.1b monophyletic	0
3	Viridiplantae, Group I.1a, and Group I.1b monophyletic	0
4	Viridiplantae and Group I.1a monophyletic	0
5	Viridiplantae and Group I.1b monophyletic	0.022

To reconcile this observation with those from the Fer domain (see above), the most parsimonious hypothesis would be that homologous replacements of the J domain occurred twice in Thaumarchaeota after their acquisition of the DnaJ-Fer protein from Viridiplantae (Figure 4B). Such independent homologous replacements could also explain the structural differences observed between the sequences of Viridiplantae and Thaumarchaeota, namely the presence of different central regions separating the J and the Fer domains (large in group I.1a Thaumarchaeota, short in group I.1b Thaumarchaeota, and its absence in Viridiplantae, see above).

### The complex evolutionary history of the DnaK/DnaJ/GrpE system in Archaea

Two of the three DnaJ-Fer proteins of *C. reinhardtii* (CDJ3 and CDJ4) have been shown to interact with the chloroplast Hsp70B proteins [24]. These proteins together with their partners, the co-chaperone DnaJ and the nucleotide exchange factor GrpE, are widely distributed in eukaryotes and also in bacteria (where they are encoded in a gene cluster). In contrast, in Archaea, they were initially reported only in lineages of mesophilic and thermophilic euryarchaeota [16,17]. However, at that time the available complete genome sequences were far from covering the whole diversity of the archaeal phyla [25] and many major lineages were not represented. Our survey of about a hundred archaeal genomes now available allowed us confirming the presence of the three genes in all members of the lineages where they were initially reported (Methanobacteriales, Thermoplasmatales, Halobacteriales and Methanosarcinales, Additional file 1) [16,17]. In addition, we also found them in many other major lineages, such as Thaumarchaeota, ‘Aigarchaeota’, ARMAN group, DHEV2 group, Nanohaloarchaeales, Methanomicrobiales, and Methanocellales (Additional file 1: Table S1). This increased considerably the diversity of archaea harboring the DnaK system. Worth noting, all these archaea were either mesophilic or thermophilic organisms, underlying the absence of the DnaK/DnaJ/GrpE system in hyperthermophilic archaea. In fact, to the noticeable exception of mesophilic Methanococcales for which we identified DnaK and GrpE homologues only in *Methanococcus vannielii* SB, which were not included in our phylogenetic trees because of their extreme sequence divergence, all mesophilic and thermophilic archaea encoded these three genes and, in most of these archaeal genomes, the three genes were clustered together as occurs in Bacteria (Additional file 1).

**Figure 5:**
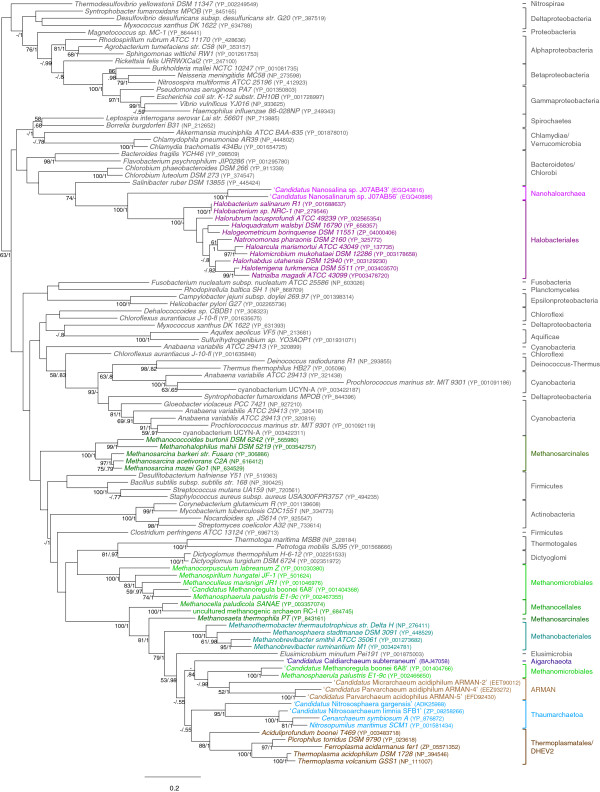
**Unrooted ML tree of the DnaK protein. **The tree was inferred with TreeFinder with the LG + Γ4 model (107 sequences and 444 positions). Numbers at nodes represent bootstrap values (100 replicates of the original alignment) and Bayesian posterior probabilities computed with TreeFinder and MrBayes, respectively (only values >50% and 0.5 are shown, dashes indicate that the corresponding support is inferior to the threshold, whereas when both supports are inferior to the thresholds no support values are indicated). The scale bar represents the average number of substitutions per site.

In agreement with previous studies [13,14,16,17] our phylogenetic analysis of a subset of 136 sequences representative of the genetic diversity of bacterial, archaeal and eukaryotic DnaK/Hsp70 sequences from complete genomes supported a clear separation between eukaryotic and prokaryotic sequences (BV = 100% and PP = 1.00), and the grouping of mitochondrial and chloroplast sequences with Alphaproteobacteria (BV < 50% and PP < 0.50) and Cyanobacteria (BV = 60% and PP = 1.00), respectively (Additional file 2). By contrast, bacterial and archaeal sequences did not form two separated monophyletic groups but appeared intricately mixed, suggesting that HGT occurred between these two domains of life. To increase the resolution of the evolutionary relationships among prokaryotic DnaK proteins, we reanalysed this dataset after removing the eukaryotic sequences (Figure 5). The monophyly of most bacterial phyla was recovered, often with strong statistical support: Aquificae (BV = 100%; PP = 1.00); Cyanobacteria (BV = 81%; PP = 1.00, a second copy that groups with Deinococcus/Thermus exists in some Cyanobacteria); Actinobacteria (BV = 100%; PP = 1.00); Thermotogae (BV = 100%; PP = 1.00); Dictyoglomi (BV = 100%; PP = 1.00); Deinococcus/Thermus (BV = 98%; PP = 0.82); Spirochaetes (BV = 58%; PP = 0.68); Chlamydiae and Verrucomicrobia (BV < 50%; PP = 0.78); Alpha-, Beta-, Gamma-, Deltaproteobacteria (BV < 50%; PP = 1.00). Similarly, the monophyly of most archaeal orders and classes harbouring a DnaK gene was recovered with high support: Halobacteria and Nanohaloarchaea (BV = 100% ; PP = 1.00); Methanosarcinales (except *Methanosaeta thermophila*, BV = 100%; PP = 1.00); Methanomicrobiales (BV = 100%; PP = 1.00); Methanobacteriales (BV = 100%; PP = 1.00); Methanocellales (BV = 100%; PP = 1.00); ARMAN (BV = 52%; PP = 1.00); Thermoplasmatales (BV = 100%; PP = 1.00) together with *Aciduliprofundum boonei* (BV = 88%; PP = 1.00) and Thaumarchaeota (BV = 95%; PP = 1.00). This indicated the ancestral presence of DnaK in these groups (i.e. prior to their diversification) and that very few HGTs among them occurred after their diversification.

As in most molecular phylogenies, the relationships among bacterial phyla remained mostly unresolved (BV < 50% and PP < 0.95, Figure 5). However, the ancestral presence of DnaK in most of them suggested that this protein was present in the last common ancestor of bacteria. By contrast, the relationships among archaeal orders and classes were well resolved but in strong contradiction with the reference species phylogeny of this domain [25]. To assess the robustness of this contradiction, we tested if the reference archaeal phylogeny was significantly rejected by the archaeal DnaK dataset. For that, the archaeal relationships observed in the DnaK ML tree were compared to those of the archaeal species reference phylogeny [25]. The AU test indicated that the DnaK dataset strongly rejected the reference phylogeny (p = 0.0). In agreement with previous proposals [16,17], this supported the hypothesis that DnaK was not present in the ancestor of Archaea and that it was acquired secondarily by some members of this domain by HGT from bacteria. A careful examination of the DnaK trees suggested that at least two independent inter-domain HGT events occurred: one to the ancestor of Halobacteria and Nanohaloarchaea and another to the ancestor of Class II methanogens (i.e., Methanosarcinales, Methanomicrobiales and Methanocellales [25]). However, the phylogeny of DnaK was not resolved enough to determine without ambiguities the bacterial donors at the origin of these HGT even if, as previously proposed [17], Firmicutes may be a possible donor in the case of the HGT to methanogenic archaea. The initial acquisitions were probably followed by secondary HGTs to and among Methanobacteriales, ARMAN, Thermoplasmatales + DHEV2, ‘*Ca*. Caldiarchaeum subterraneum’ and Thaumarchaeota. Worth noting, the two latter lineages did not form a monophyletic group (Figure 5), in contradiction with the expected species phylogeny [25]. This suggests two independent acquisitions of the gene coding for DnaK from two different euryarchaeotal donors, but the statistical support for the corresponding branches are too low to reach a definitive conclusion. Interestingly, if inter-domain HGTs from Bacteria to Archaea were clearly supported by our analyses, at least one HGT occurred in the opposite direction. This concerned the DnaK of *Elusimicrobium minutum* (Class Elusimicrobia, previously referred as Termite Group 1), which was nested among archaeal sequences with strong support (Figure 5). This ultramicrobacterium was isolated from humivorous beetle larvae and some close relatives have been detected in gut or faeces of termites, cockroaches, and mammals such as chimpanzee, horses or cows [36], environments that are also inhabited by diverse methanogenic archaea. Accordingly, HGT among those microorganisms is not unexpected.

The phylogenies of GrpE and DnaJ were less resolved than that of DnaK, in particular for the deepest nodes (Additional file 3 and Additional file 4), as expected from the smaller number of conserved positions that could be kept for phylogenetic analyses (105 and 227 positions, respectively). However, despite the weaker signal, most of the monophyletic groups observed in the DnaK trees were also recovered in the GrpE and DnaJ phylogenies, which suggested that the three proteins have undergone similar evolutionary histories. This agreed with the fact that the corresponding genes are clustered in most prokaryotic genomes (Additional file 1).

## Discussion

The unexpected discovery eight years ago of an atypical protein composed of Ferredoxin domain associated to a J domain in the marine archaeal fosmid EC-39 led to the proposal that Thaumarchaeota should have a DnaK protein [18]. This prediction was confirmed a few years later after the identification of genes coding for the DnaK/DnaJ/GrpE system in complete genome sequences of representatives of this phylum [37]. Our phylogenomic analysis showed that the DnaJ-Fer and the DnaK/DnaJ/GrpE proteins have two different origins in this archaeal phylum. The thaumarchaeotal DnaJ-Fer protein resulted from a complex history involving at least two inter-domain HGTs: from Cyanobacteria to Viridiplantae and then from Viridiplantae to Thaumarchaeota, in addition to two independent replacements of the original viridiplantae J domain by J domains of unknown bacterial origin during the diversification of Thaumarchaeota (Figure 3B). By contrast, the phylogenetic analysis of the DnaK, DnaJ and GrpE proteins suggested that these proteins were acquired by the ancestor of Thaumarchaeota by HGT from an unidentified euryarchaeotal donor. The thaumarchaeotal DnaK/DnaJ/GrpE and DnaJ-Fer form therefore a complex chimera, mixing components from bacterial, euryarchaeotal and eukaryotic origin. Identifying the precise functional role of these proteins in Thaumarchaeota will require further investigation.

Due to its large distribution in present day organisms, DnaK was initially proposed as a good phylogenetic marker to infer ancient phylogenies and, more precisely, to decipher the relationships among the three domains of life and their main phyla [38-40]. In particular, the presence of a 23 amino acids deletion shared by Actinobacteria and Firmicutes (referred as Monoderma) and Archaea, but not by Gram negative bacteria (referred as Diderma) and Eucarya was interpreted as the evidence that Archaea derived from Gram positive bacteria [38], dismissing the hypothesis of inter-domain HGT. However, the phylogenetic analysis of DnaK (and of its two partners DnaJ and GrpE) showed later that the evolutionary history of these proteins has been largely affected by HGT. In particular, the strong discrepancies observed between the phylogeny of archaeal DnaK and the species tree indicates that multiple HGTs are responsible of the taxonomic distribution of DnaK in Archaea [17,41]. Thus, the deletion detected by Gupta (which now has been shown to be present also in Thermotogae, Dictyoglomi and Fusobacteria) should not be interpreted as relevant for species phylogeny but just as a strong signature for the gene transfers mentioned above. Therefore, DnaK appears not to be a reliable marker to infer ancient evolutionary relationships, as already suggested in previous works [41].

Phylogenetic and molecular analyses have shown that adaptation to mesophily occurred several times independently during the diversification of Archaea [22,42,43]. Because, DnaK is an important heat shock chaperone involved, among others, in thermal stresses [1], we have postulated previously that its acquisition could have help for the adaptation of archaeal hyperthermophiles to mesophilic environments [18]. Strengthening this hypothesis, DnaK, DnaJ and GrpE are found only in thermophilic and mesophilic archaea which have acquired these genes several times independently, either from bacteria through inter-domain HGTs or from archaea already adapted to mesophilic environments. However, the acquisition of DnaK cannot be considered as obligatory for that adaptation. For example, in the case of Methanococcales, we identified an atypical DnaK gene in *Methanococcus vannielii* SB, but we did not detect it in other mesophilic members of this archaeal order. This illustrated the fact that additional contributing factors, such as protein amino acid composition, are surely important to determine the optimal growth temperature of microorganisms. For instance, always among the Methanococcales, the mesophilic *Methanococcus maripaludis* has been shown to harbour amino acid signatures typical of thermophilic or hyperthermophilic organisms [44], making tempting to speculate that the proteins of this archaeon are intrinsically resistant to heat shocks and, thus, that heat shock chaperones are dispensable in this organism.

Beside those aspects, the evolutionary history of the DnaJ-Fer protein provided an interesting temporal landmark between the Eucarya and Archaea domains. Indeed, the association between the Fer and the J domains composing this protein very likely occurred in an ancestor of the Viridiplantae, before their diversification but after their divergence from the two other Archaeplastida lineages (i.e. the Glaucophyta and the Rhodophyta, which do not have this fused protein) (Figure 3A). Then, the resulting gene was transferred to the ancestor of Thaumarchaeota groups I.1a and I.1b (Figure 3B), more precisely before the divergence of these two lineages but likely after their separation from the HWCG III group (Figure 3A). This indicated that the divergence of the groups I.1a and I.1b is more recent than the divergence between Viridiplantae and the two other Archaeplastida lineages but more ancient than the diversification of Viridiplantae. This illustrates how HGTs can be useful to date evolutionary events relatively against each other [45]. According to fossil record and molecular dating estimates, the divergence of Viridiplantae from the two other Archaeplastida lineages occurred ~950 million years ago whereas the diversification of Viridiplantae started ~750 million years ago [46]. The HGT from Viridiplantae to Thaumarchaeota occurred most likely during this time window, so the divergence of the groups I.1a and I.1b Thaumarchaeota and their diversification could be less than ~950 million years old.

## Conclusions

Phylogenomic analysis supports that the proteins DnaK, DnaJ, GrpE and DnaJ-Fer have a chimerical origin in Thaumarchaeota, which acquired them by HGT from different donors, including bacterial and eukaryotic species. Similar HGT events have occurred independently in other archaeal groups. This suggests that the acquisition of these proteins has probably played an important role in the convergent adaptation of these archaea to mesophilic and thermophilic lifestyles from their hyperthermophilic ancestors. In addition, these HGT events can be used as markers for the relative dating of the diversification of donor and acceptor groups as, for example, the Thaumarchaeota, which have received their DnaJ-Fer protein from Archaeplastida.

## Methods

### Dataset assembly

The DnaJ-Fer protein homologues were retrieved from the non-redundant (nr) and the environmental databases at the NCBI (http://www.ncbi.nlm.nih.gov) with the BlastP program (default parameters) [47] using as seeds the DnaJ-Fer protein from the uncultured archaeon DeepAnt-EC39 fosmid (AY316120.1), and the sequences of *Chlamydomonas reinhardtii* CDJ3, 4 and 5 (XP_001700257.1, XP_001699768.1 and XP_001700843.1 respectively). The DnaJ-Fer sequence from *Nitrososphaera viennensis* was kindly provided by C. Schleper and A. Spang. To ensure the exhaustive retrieval of eukaryote sequences we queried EST and ongoing genome project databases: the JGI (http://genome.jgi-psf.org/) for *Ostreococcus* sp. RCC809, *Emiliania huxleyi*, *Chlorella vulgaris* and *Chlorella* sp. NC64A; the TIGR (http://plantta.jcvi.org/) for *Pinus taeda*; the *Cyanidioschyzon merolae* Genome Project (http://merolae.biol.s.u-tokyo.ac.jp/), and the *Galdieria sulphuraria* Genome Project (http://genomics.msu.edu/galdieria/about.html). The absence of DnaJ-Fer homologues in any archaeal or eukaryotic complete genome was verified by tBlastN searches against the corresponding nucleic acid sequence. The presence of the J and the Fer domains in the retrieved homologues was systematically verified using Pfam (Pfam profiles PF00226 and PF13459, respectively). The J and Fer domains were then analysed separately using the same strategy as previously to retrieve proteins containing these domains.

DnaK, GrpE and DnaJ homologues were retrieved from 92 archaeal complete genome sequences available at NCBI with BlastP (default parameters) using the sequences from *N. maritimus* as seeds (YP_001581434, YP_001581433.1 and YP_001581435, respectively). The absence of homologues in any genome was systematically verified by tBlastN searches against the corresponding nucleic acid sequence. Eukaryotic and bacterial homologues were retrieved from a subset of four and 86 complete genomes representative of the taxonomic diversity of these two domains using BlastP (default parameters). In the case of DnaJ, we checked the domain composition of the retrieved homologues with PFAM in order to distinguish *bona fide* DnaJ proteins (harbouring a J-domain (PF00226), the cysteine rich central domain (PF00684) and the C-terminal domain (PF01556)) from other J-proteins.

We thus obtained six different sequence datasets, and we tested various programs to align them, including (Mafft v6.833b [48], Probcons v1.12 [49], and Muscle v3.6 [50]). The quality of the resulting alignments was visually inspected in order to keep those for which the residues of the conserved domains were correctly aligned. Probcons provided better results for the DnaJ-Fer, the J domain and the Fer domain datasets, whereas Mafft provided better results in the case of the DnaK, GrpE and DnaJ datasets. The selected alignments were edited and manually refined with the program ED of the MUST package [51]. The regions where the alignment was ambiguous were removed using the NET program from the MUST package.

### Phylogenetic reconstruction

The DnaJ-Fer, DnaK, GrpE and DnaJ alignments were analysed by maximum likelihood (ML) and Bayesian approaches (BI). For each dataset, the LG model was proposed as the best suited evolutionary model according to the "propose model tool" of TreeFinder v2011 [52] with the AICc criterion. Alternative models (e.g. WAG, JTT, etc.) were also tested. The resulting trees were consistent with those inferred with the LG model (not shown).

ML tree reconstructions were performed using PhyML v3.0 [53] and TreeFinder v2011 [52]. The robustness of the resulting trees was estimated by the non-parametric procedure implemented in PhyML and TreeFinder (100 replicates of the original dataset). The resulting trees were very similar, so we decided to show only the ML trees inferred with TreeFinder.

BI of DnaJ-Fer, DnaK, DnaJ and GrpE proteins was carried out with PhyloBayes v 3.3 with the LG model and a gamma distribution of substitution rates with four categories [54]. Phylobayes was run with four independent chains for at least 10,000 cycles, saving one tree in ten. The first 300 trees were discarded as "burn-in", and the remaining trees from each chain were used to test for convergence and compute the 50% majority rule consensus tree. In the case of DnaK, the chains did not converge even after 10,000 cycles. Therefore, BI trees for this marker were computed with MrBayes v.3.0b4 [55] with a mixed substitution model and a Gamma distribution of substitution rates with 4 categories. Searches were run with 4 chains of 1,000,000 generations for which the first 1,000 generations were discarded as “burn in”, trees being sampled every 100 generations.

The analyses of the J and Fer domains were divided in two steps. First, all the homologous sequences were analysed by neighbor-joining (NJ) using the MUST package [51]. Based on this preliminary phylogenetic tree, we selected the closest homologues of the DeepAnt-EC39 fosmid and *C. reinhardtii* sequences and a subset of more distantly related homologues representative of the genetic diversity of these domains. These sequences were used to carry out ML and BI analysis with TreeFinder, PhyML and PhyloBayes as previously described.

The comparison of different tree topologies was done by applying the Approximately Unbiased test [56] implemented in TreeFinder with the same evolutionary models and parameters as for ML phylogenetic inference.

## Competing interests

The authors have declared that no competing interests exist.

## Authors’ contributions

CB-A and DM conceived this study. CP, CB-A and DM designed and carried out the phylogenetic analyses. CP, CB-A, PL-G and DM wrote the manuscript. All authors read and approved the final manuscript.

## Supplementary Material

Additional file 1 **Table showing the taxonomic distribution of DnaJ-Fer, DnaK, DnaJ and GrpE proteins in Archaea. **Numbers correspond to accession numbers in the NCBI Genpep database in the 92 complete genomes available in July 2011 and that of, '*Ca. *Nitrosoarchaeum koreensis', a thaumarchaeotal genome available more recently. The two divergent sequences of DnaK and GrpE found in *Methanococcus vannielii *SB are underlined. Click here for file

Additional file 2 **Unrooted ML tree of the DnaK protein (136 sequences and 444 positions) inferred with TreeFinder and the LG + Γ4 model. **Numbers at nodes represent bootstrap values and Bayesian posterior probabilities computed with TreeFinder and MrBayes, respectively (only values >50% and 0.5 are shown, dashes indicate the corresponding support is inferior to the threshold, whereas when both supports are inferior to the thresholds no support values are indicated). Archaeal sequences are shown with colours according to their taxonomic classification. The scale bar represents the average number of substitutions per site. Click here for file

Additional file 3 **Unrooted ML tree of the DnaJ protein (102 sequences and 227 positions) inferred with TreeFinder and the LG + Γ4. **Numbers at nodes represent bootstrap values and Bayesian posterior probabilities computed with TreeFinder and MrBayes, respectively (only values >50% and 0.5 are shown, dashes indicate that the corresponding support is inferior to the threshold, whereas when both supports are inferior to the thresholds no support values are indicated). Archaeal sequences are shown with colours according to their taxonomic classification. The scale bar represents the average number of substitutions per site. Click here for file

Additional file 4 **Unrooted ML tree of the GrpE protein (101 sequences and 105 positions) inferred with TreeFinder and the LG + Γ4. **Numbers at nodes represent bootstrap values and Bayesian posterior probabilities computed with TreeFinder and MrBayes, respectively (only values >50% and 0.5 are shown, dashes indicate the corresponding value is inferior to the threshold, whereas when both supports are inferior to the thresholds no support values are indicated). Archaeal sequences are shown with colours according to their taxonomic classification. The scale bar represents the average number of substitutions per site. Click here for file
