# Supplementary figures and images for: Horizontal gene transfer of a chloroplast DnaJ-Fer protein to Thaumarchaeota and the evolutionary history of the DnaK chaperone system in Archaea
Source: BMC Evol Biol. 2012 Nov 26;12:226. doi: 10.1186/1471-2148-12-226 (PMC3564930; doi:10.1186/1471-2148-12-226)

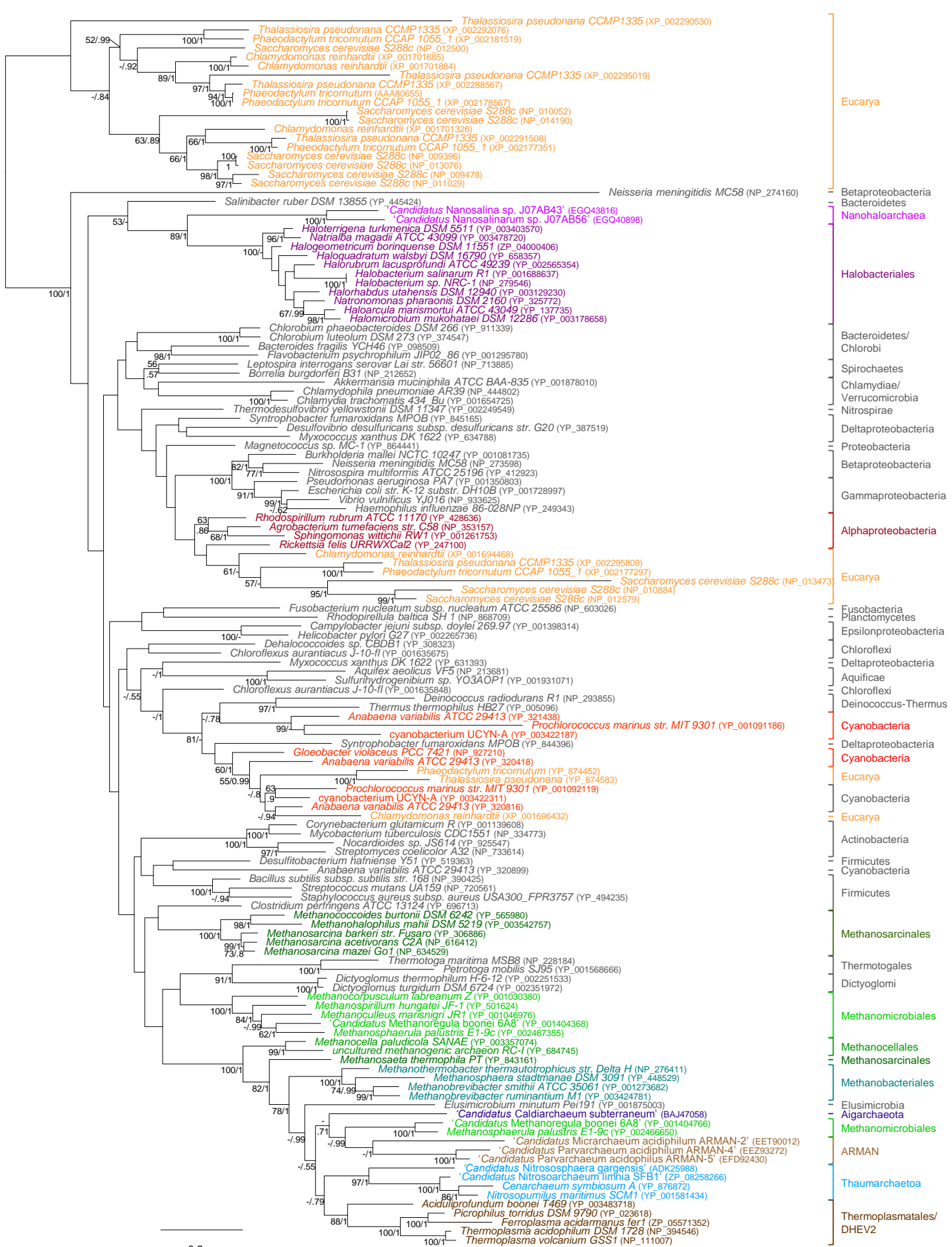

Supplement: Additional file 2 — Unrooted ML tree of the DnaK protein (136 sequences and 444 positions) inferred with TreeFinder and the LG + Γ4 model. Numbers at nodes represent bootstrap values and Bayesian posterior probabilities computed with TreeFinder and MrBayes, respectively (only values >50% and 0.5 are shown, dashes indicate the corresponding support is inferior to the threshold, whereas when both supports are inferior to the thresholds no support values are indicated). Archaeal sequences are shown with colours according to their taxonomic classification. The scale bar represents the average number of substitutions per site. [file 1471-2148-12-226-S2.pdf]

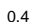

Supplement: Additional file 3 — Unrooted ML tree of the DnaJ protein (102 sequences and 227 positions) inferred with TreeFinder and the LG + Γ4. Numbers at nodes represent bootstrap values and Bayesian posterior probabilities computed with TreeFinder and MrBayes, respectively (only values >50% and 0.5 are shown, dashes indicate that the corresponding support is inferior to the threshold, whereas when both supports are inferior to the thresholds no support values are indicated). Archaeal sequences are shown with colours according to their taxonomic classification. The scale bar represents the average number of substitutions per site. [file 1471-2148-12-226-S3.pdf]

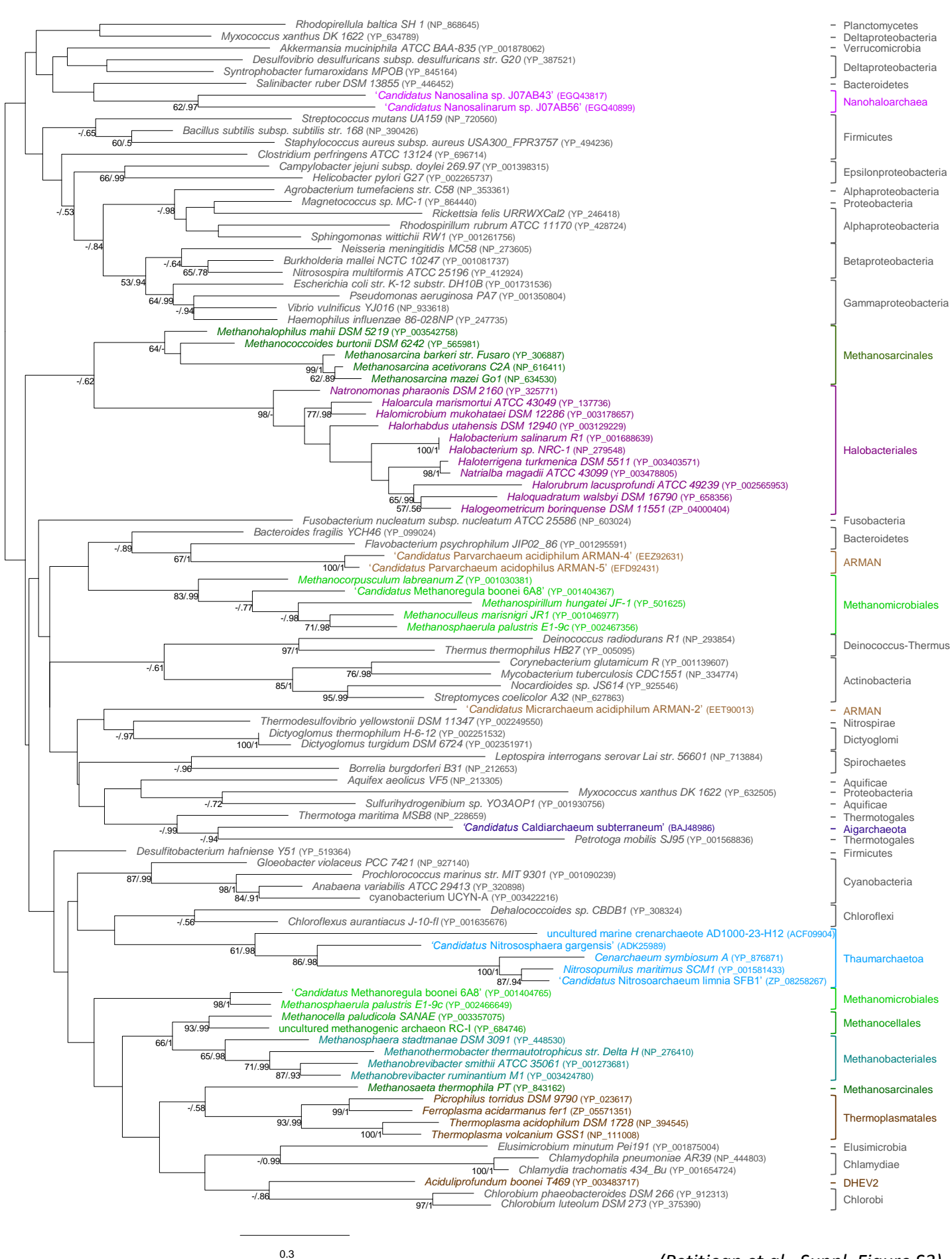

Supplement: Additional file 4 — Unrooted ML tree of the GrpE protein (101 sequences and 105 positions) inferred with TreeFinder and the LG + Γ4. Numbers at nodes represent bootstrap values and Bayesian posterior probabilities computed with TreeFinder and MrBayes, respectively (only values >50% and 0.5 are shown, dashes indicate the corresponding value is inferior to the threshold, whereas when both supports are inferior to the thresholds no support values are indicated). Archaeal sequences are shown with colours according to their taxonomic classification. The scale bar represents the average number of substitutions per site. [file 1471-2148-12-226-S4.pdf]
